# Small RNA modifications: regulatory molecules and potential applications

**DOI:** 10.1186/s13045-023-01466-w

**Published:** 2023-06-22

**Authors:** Qunli Xiong, Yaguang Zhang

**Affiliations:** 1 State Key Laboratory of Biotherapy and Cancer Center, National Clinical Research Center for Geriatrics and Frontiers Science Center for Disease-Related Molecular Network, West China Hospital, Sichuan University, Chengdu, 610041 People’s Republic of China; 2 Abdominal Oncology Ward, Cancer Center, West China Hospital, Sichuan University, Chengdu, China

**Keywords:** Small RNA, RNA modifications, MicroRNA, PIWI-interacting RNA, tRNA-derived small RNA, N6-methyladenosine, 2′-O-methylation, 5-Methylcytosine, Pseudouridine

## Abstract

Small RNAs (also referred to as small noncoding RNAs, sncRNA) are defined as polymeric ribonucleic acid molecules that are less than 200 nucleotides in length and serve a variety of essential functions within cells. Small RNA species include microRNA (miRNA), PIWI-interacting RNA (piRNA), small interfering RNA (siRNA), tRNA-derived small RNA (tsRNA), etc. Current evidence suggest that small RNAs can also have diverse modifications to their nucleotide composition that affect their stability as well as their capacity for nuclear export, and these modifications are relevant to their capacity to drive molecular signaling processes relevant to biogenesis, cell proliferation and differentiation. In this review, we highlight the molecular characteristics and cellular functions of small RNA and their modifications, as well as current techniques for their reliable detection. We also discuss how small RNA modifications may be relevant to the clinical applications for the diagnosis and treatment of human health conditions such as cancer.

## Introduction

**Fig. 1:**
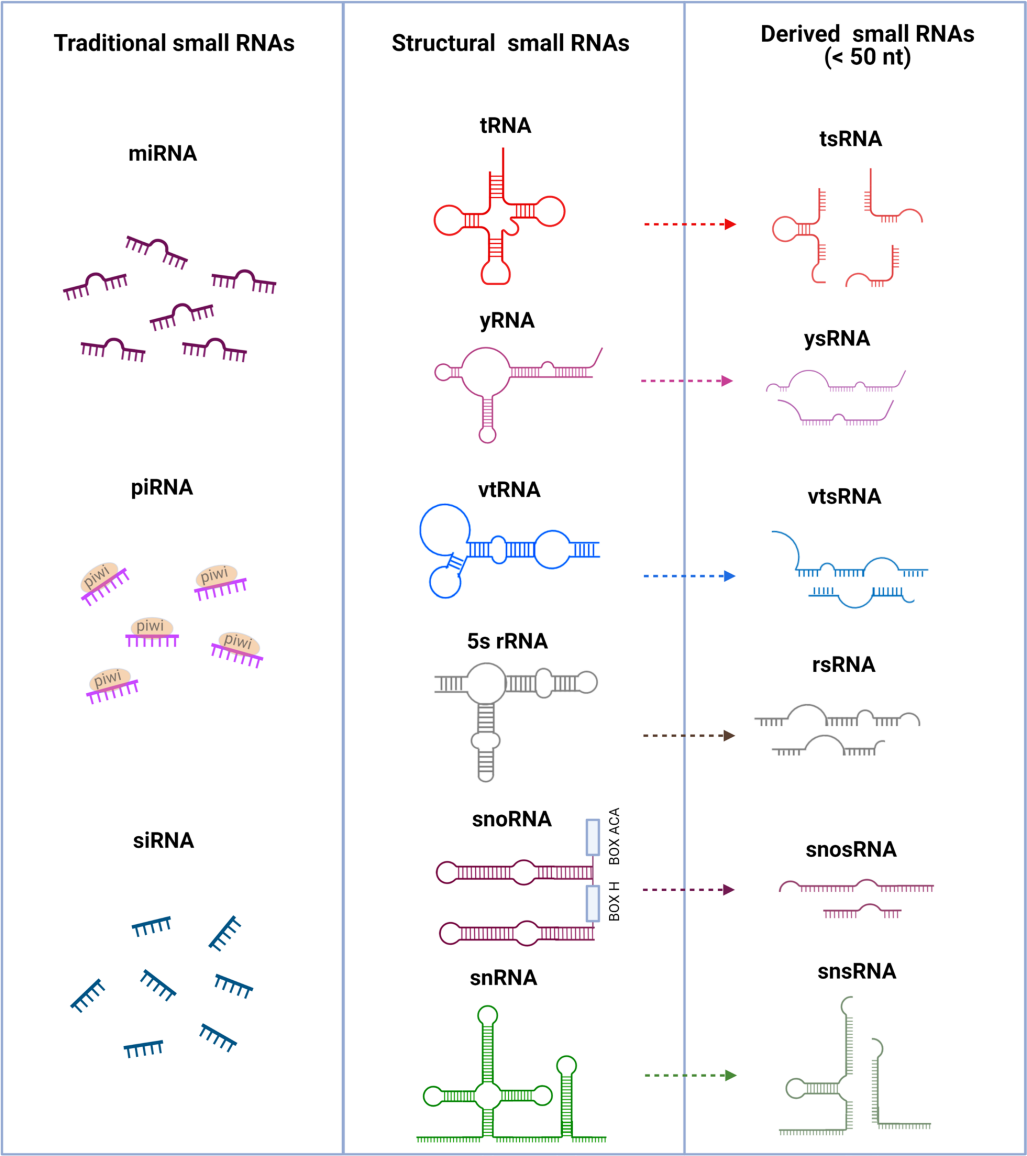
The structure and classification of small RNAs. The left panel displays traditional small RNAs, including miRNA, piRNA and siRNA; the middle panel displays structural small RNAs, including tRNA, yRNA, vtRNA, rRNA (containing 5s rRNA and 5.8s rRNA, and 5s rRNA was showed here), snoRNA and snRNA; the right panel displays derived small RNAs, whose fragment sizes are less than 50nt, and predominantly includes tsRNA from tRNA, ysRNA from yRNA, vtsRNA from vtRNA, rsRNA from 5s rRNA, snosRNA from snoRNA and snsRNA from snRNA. This figure was developed using BioRender.com

RNA molecules play essential and diverse roles in numerous biological functions, as studied in organisms ranging from prokaryotes to eukaryotes [1–3]. From those studies, it emerged that post-transcriptional modifications are essential for the functions of RNA molecules to carry out their cellular functions. In the 1960s, scientists first discovered modifications in RNA bases through enzymatic digestion and electrophoresis [4]. Since then, over 170 different types of RNA post-transcriptional chemical modifications have been described across all currently known RNA species [5]. Over the course of these investigations, the enzymes responsible for writing (catalyzing and modifying nucleotides), reading (recognizing and binding modified nucleotides) and erasing (catalyzing the removal of specific modifications) RNA modifications have also been discovered [6–9]. Small RNAs, which are a class of noncoding RNAs that are less than 200 nucleotides in length, are widely present in various cell types and tissues [10–12]. Over the past 20 years, extensive research has led to their classification on the basis of their size and structural characteristics, as follows: traditional small RNAs, structural small RNAs and derived small RNAs (also called non-canonical small RNAs, Fig. 1 and Table 1) [13]. These small RNAs are involved in various biological processes through different mechanisms. For example, traditional small RNA species, including, microRNA (miRNA), PIWI-interacting RNA (piRNA) and small interfering RNA (siRNA), interact with Argonaute proteins to mediate RNA-silencing effects. Furthermore, structural small RNAs (including tRNA, rRNA, snoRNA, snRNA, yRNA and vtRNA) are essential components within cells that regulate physiological homeostasis. In contrast, non-canonical small RNAs represent structural RNAs of poorly characterized functions independent of Argonaute proteins, and these are generated following enzymatic cleavage by evolutionarily ancient RNases [14]. Further to these small RNAs, new evidence suggests that small RNAs can be modified in a variety of ways which significantly influence their functions across various biological processes [14, 15]. Here, we detail the roles for small RNA modifications in the biogenesis and functions of small RNAs, with a focus on the following modifications: N6-methyladenosine (m6A), 2′-O-methylation (Nm), 5-methylcytosine (m5C) and pseudouridine (Ψ). Also, we summarize the current methods of detecting these small RNAs, highlight the evidence for this molecular process in cell and tissue homeostasis and discuss the potential clinical application of small RNAs and its modifications in human disease.

**Table 1:** The characteristics of different types of small RNAs

Classification	Length (nt)	Precursor	Processing	Function	References
Micro RNA(miRNA)	~ 20–25	Hairpin loop-like precursor miRNA	transcribed by RNA pol II, cleaved by Drosha/DGCR8 and Dicer	Binds to Argonaute protein and forms a RISC complex that participates in transcriptional gene silencing, inhibition of mRNA translation and in mRNA decay	[16, 17]
PIWI-interacting RNA(piRNA)	~ 18–30	Long single-stranded RNA	transcribed by RNA pol II, processing by primary procession pathway and ping-pong cycle by Zuc, Aub, AGO3	Interacts with PIWI proteins to regulate gene silencing in a miRNA-like manner and regulates germ cell genome stability through recruitment of epigenetic regulators	[18, 19]
Small interfering RNA(siRNA)	~ 20–25	Long double-stranded RNA	transcribed by RNA pol II, processing by Dicer to cleavage	Binds to Argonaute protein and forms a RISC complex that participates in mediating mRNA decay	[10, 20]
Transfer RNA(tRNA)	~ 74–93	Precursor tRNA	transcribed by RNA pol III, processing by Ribonuclease P, Ribonuclease Z,endonuclease nucleotidyl transferase to cleavage and modify	Transports amino acids to the ribosome to synthesize polypeptide chains	[21, 22]
Small nuclear RNA (snRNA)	~ 90–200	\	Transcripted by RNA polymerase III and II	Combines with proteins to form small nuclear ribonucleoproteins (snRNPs) involved in pre-mRNA splicing	[23]
Small nucleolar RNA (snoRNA)	~ 60–400	\	Transcribed by RNA polymerase II, processing by ribonuclease	Binds to specific proteins to form small nucleolar ribonucleoprotein (snoRNPs) that regulate the modification of ribosomal RNA (rRNA)	[24]
5S RNA	120	pre-5S RNA	Transcribed by RNA polymerase III	Binds to ribosomal protein L5 to form a stable complex that is involved in the assembly of the larger ribosomal subunits	[25]
Vault RNA (vtRNA)	~ 88–140	\	Transcribed by RNA polymerase III	Binds to Vault proteins and form complexes; functions remain poorly characterized	[26, 27]
Y RNA	~ 80–110	\	Transcribed by RNA polymerase III, processing by ribonuclease	Binds to Ro60 protein and La protein to form Ro-RNP (Ro60 containing ribonucleoprotein) complex which regulates RNA stability, cellular stress responses, as well as initiation of chromosomal DNA replication and Ro60 protein activation	[28, 29]
Transfer RNA-derived small RNA (tsRNA)	~ 15–40	tRNA	Cleaved by Angiogenin, Dicer, RNase T2, RNase Z, ELAC2, Rnylp, etc	Functions by mimicry or displacement of tRNA; forms RNP complexes; binds to Argonaute	[30, 31]
Y RNA derived small RNA (ysRNA)	~ 22–25 and ~ 27–36	Y RNA	May cleaved by Caspase or RNsase1	Function is unclear; abundant in extracellular spaces, such as in serum, plasma and other biofluids	[32–34]
vtRNA derived small RNA (vtsRNA)	~ 23–32	vtRNA	Dicer	Function is unclear; predicted to function in a miRNA-like manner	[35, 36]
ribosomal RNA derived small RNA (rsRNA)	~ 21–44	rRNA	Unclear	Function is unclear; enriched in mature mouse sperm; present in human peripheral blood serum; its presence is linked with inflammation in humans	[34, 37]
Small nucleolar RNA derived small RNA (snosRNA)	17–19; 20–24; > 26	snoRNA	Unclear	Function is unknown; likely resembles miRNA; can associate with argonaute proteins and influence translation; longer snosRNAs may form complexes with hnRNPs and influence gene expression	[38, 39]

## Small RNA modifications

### N6-methyladenosine (m6A)

**Fig. 2:**
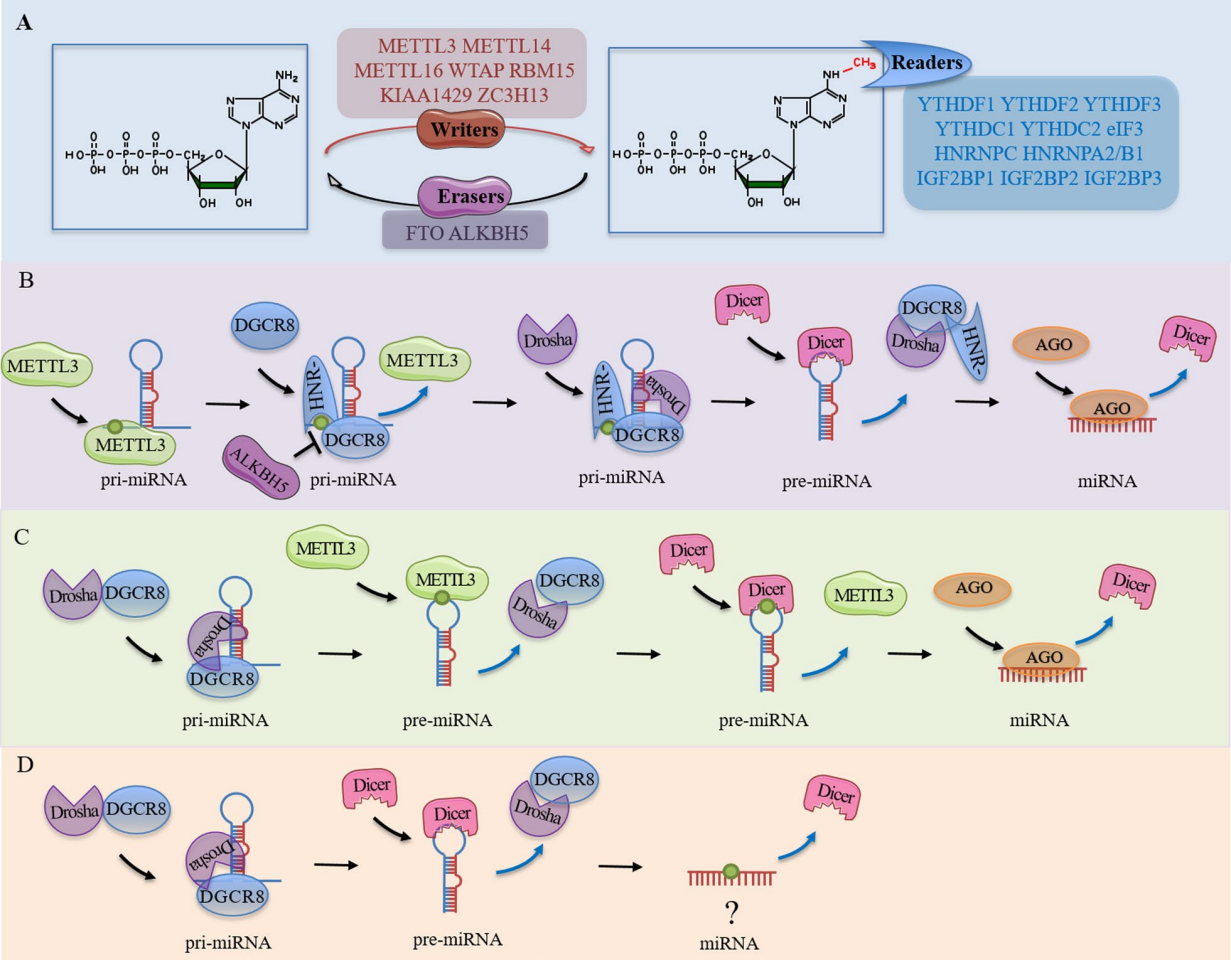
The m6A modification in miRNA. A. The chemical structure of adenosine and the site of methylation on N6 are shown alongside the enzymes (writers, eraser and readers) known to be involved. B. The m6A modification of pri-miRNA mediated by METTL3 or METTL14 is recognized by HNRNPA2/B1, so as to promote the interaction between DGCR8 and pri-miRNA, leading to acceleration of miRNA biosynthesis. On the contrary, ALKBH15 demethylates pri-miRNA, preventing DGCR8 from interacting with pri-miRNA, and this results in blockade of mature miRNA synthesis. HNR-: HNRNPA2/B1. C. The m6A modification in pre-miRNA mediated by METTL3 promotes the binding of Dicer to pre-miRNA which, in turn, accelerates the biosynthesis of miRNA (note that the specific m6A modification sites remain unknown). D. While studies have found that m6A modifications can be detected on mature miRNAs, the origin and biological relevance of such modifications remains to be better characterized

N6-methyladenosine (m6A), first discovered in the 1970s [40, 41], is a methylation modification of the sixth nitrogen (N) atom of adenine (A). The m6A modification is one of the most abundant type of modification to messenger RNAs (mRNAs) in the biological world [42, 43], including in small RNAs of eukaryotic species [44]. This m6A modification can be catalyzed by S-adenosylmethionine (SAM) binding proteins methyltransferase-like 3 (METTL3) and methyltransferase-like 14 (METTL14) [45, 46]. Notably, other cofactors, such as Wilms tumor-associating protein (WTAP) [47], methyltransferase-like 16 (METTL16) [48], RNA-binding motif protein 15 (RBM15) [49], KIAA1429 (also called VIRMA) and zinc finger CCCH domain-containing protein 13 (ZC3H13) [50], are also known to be essential for catalyzing function of m6A methyltransferases. The proteins known as fat mass and obesity-associated protein (FTO) and ALKB homolog 5 (ALKBH5) are both also identified as m6A demethylases [51, 52]. On the other hand, members of YT521-B homology domain family 1/2/3 (YTHDF1/2/3) [7, 53], YT521-B homology domain-containing proteins 1/2 (YTHDC1/2) [7, 54], members of the heterogeneous nuclear ribonucleoprotein protein families (including HNRNPC, HNRNPA2/B1) [7, 55], eukaryotic translation initiation factor 3 (eIF3) [49], as well as insulin-like growth factor-2 mRNA-binding proteins 1/2/3 (IGF2BP1/2/3) [56] have all been characterized as reader proteins that recognize m6A methylation (Fig. 2A).

In 2015, Alarcon and colleagues reported that alterations to levels of the methyltransferase METTL3 affected the expression of mature miRNA as well as unprocessed primary miRNA (pri-miRNA), in addition to its known effect on mRNAs [57]. This result suggests that m6A is associated with miRNA biosynthesis. As described [13], the first step in miRNA biosynthesis involves binding and recognition by the double-stranded RNA-binding protein (DGCR8) to the junction between the pri-miRNA hairpin stem and the flanking single-stranded RNA within the nucleus, followed by recruitment of RNase III endonuclease Drosha to form a microprocessor complex. This leads to cleavage of pri-miRNA to produce a precursor miRNA (pre-miRNA) species. The pre-miRNA then binds exportin 5 and is transported to the cytoplasm to be cleaved into mature miRNA by Dicer. Interestingly, Alarcon and colleagues found that this biological process is dependent on m6A modification of RNA [57]. Indeed, METTL3 can methylate pri-miRNAs for HNRNPA2/B1 recognition, following which HNRNPA2/B1 recruits and interacts with DGCR8 to bind to pri-miRNA, leading to acceleration of miRNA production. Thus, m6A is an important post-transcriptional modification of efficient miRNA biosynthesis within cells (Fig. 2B) [57]. This finding provides important insight into the role of m6A in various biological processes, as well as in the progression of human diseases. For example, a mechanism for aberrant cell proliferation in bladder cancer implicates a pathway in which high METTL3 expression enhances DGCR8 recognition and binding of m6A-modified pri-miR221/222 which, in turn, potentiates miR221/222 maturation and subsequent reduction in levels of phosphatase and tensin homolog (PTEN), a known target of miR221/222 [58]. Also, in the context of patients spinal tissue degeneration, it has been reported that METTL14 regulates m6A modification of pri-miR-34a-5p to accelerate DGCR8 recognition and, through this mechanism, increases miR-34a-5p to target silent information regulator sirtuin 1 (SIRT1) and, in the process, promotes Tumor necrosis factor-alpha (TNF-α)-induced cell senescence within the nucleus pulposus invertebral disc tissue [59]. In addition to the actions of methyltransferases, m6A demethylases and reader proteins also affect the biological processes of miRNAs. For example, In lung cancer, the m6A reader HNRNPA2B1 interacts with LINC01234 to recruit DGCR8, and this leads to potentiation and accumulation of miR-106b-5p which, in turn, exerts a downregulatory effect on cryptochrome circadian regulator 2 (CRY2) levels, leading to elevated c-Myc levels and lung cancer growth [60]. In the scenario lung fibroblast activation and silica-induced lung fibrosis, it has been reported that ALKBH5, a demethylase, can demethylate pri-miR-320a-3p to prevent its interaction with DGCR8 and this, in turn, blocks the generation of miR-320a-3p, leading to dysregulation in the expression of its target genes, including forkhead box M1 (FOXM1), which ultimately leads to lung tissue damage [61]. Table 2 provides a summary of the roles of small RNA modifications in various cellular contexts in homeostasis and disease.

**Table 2:** Summary of m6A modifications to small RNAs and their cellular functions in homeostasis and human disease

Small RNA	Enzyme	Regulator network (↑ denotes upregulation, ↓ denotes downregulation)	Cellular function	Disease association	Reference
miR-375	METTL14	lncRNA UCA1 ↑ → METTL14↓ → pri-miR-375↑, miR-375↓ → SOX12↑	Promotes cell proliferation and invasion but inhibits apoptosis	Breast cancer	[70]
miR‐181b‐3p	FTO	FTO ↑→ miR‐181b‐3p ↓ → ARL5B ↑	Promotes cell migration and tissue invasion	Breast cancer	[71]
miR-221-3p	METTL3	METTL3 ↑ → m6A-modified pri-miR-221-3p ↑, miR-221-3p ↑ → HIPK2 ↓ →Che-1 ↑	Enhances drug resistance in adriamycin-resistant breast cancer cells	Breast cancer	[72]
miR-374c-5p	Unknown	Cadmium treatment → m6A-modified pri-miR-374c ↓ → pri-miR-374c ↑, miR-374c-5p ↓ → GRM3 ↑	Promotes cell proliferation and metastasis	Breast cancer	[73]
miR-1246	METTL3	METTL3 ↑ → m6A-modified pri-miR-1246 ↑ → pri-miR-1246 ↓, miR-1246 ↑ → SPRED2 ↓ → activates RAF/MEK/ERK pathway	Promotes cancer cell metastasis	Colorectal cancer	[74]
Let-7b-5p	METTL3	Metformin stimulation-METTL3 ↑ → pri-Let-7b ↓, pre-Let-7b↑, Let-7b-5p ↑ → suppresses Notch signaling	Increases sensitivity to Osimertinib therapy in patients with cancer	Lung cancer	[75]
miR-143-3p	METTL3	METTL3 ↑ → m6A-modified pre-miR-143-3p ↑ → pre-miR-143-3p ↓, miR-143-3p ↑ → VASH1 ↓	Triggers epithelial-mesenchymal transition (EMT) in a blood brain barrier model of ell invasion, as well as angiogenesis in the context of lung cancer	Lung cancer	[76]
miR-106b	HNRNPA2/B1	HNRNPA2B1 ↑ → pri-miR-106b ↓, pre-miR-106b ↑, miR-106b-5p ↑ → SFRP2 ↓ → activates Wnt/β-catenin signaling	Promotes cell stemness, proliferation, migration and tumor growth of lung adenocarcinoma cells	Lung cancer	[77]
miR-663	METTL3	METTL3 ↑ → m6A-modified pri-miR-663 ↑ → pri-miR-663 ↓, miR-663 ↑ → SOCS6 ↓	Promotes proliferation, migration and invasion of lung cancer cells	Lung cancer	[78]
miR-486	Unknown	Propofol treatment → m6A-modified pri-miR-486 ↑ → pri-miR-486 ↓, miR-486-5p ↑ → inactivates the RAP1-NF-kappaB signaling axis	Enhances cisplatin-sensitivity	Lung cancer	[79]
miR-576	FTO	FTO ↑ → m6A -modified pri-miR-576 ↓ → pri-miR-576 ↑, miR-576 ↓ → CDK6 ↑	Promotes tumor proliferation and invasion	Bladder cancer	[80]
miR221/222	METTL3	METTL3 ↑ → m6A-modified pri-miR221/222 ↑ → pri-miR221/222 ↓, miR221/222 ↑ → PTEN ↓	Promotes tumor proliferation	Bladder cancer	[81]
miR-146	METTL3	Melittin stimulation → METTL3 ↓ → m6A-modified pri-miR-146 ↓ → pri-miR-146 ↑, miR-146a-5p ↓ → regulates NUMB/NOTCH2 pathway	Induces apoptosis and inhibits tumor growth	Bladder cancer	[82]
miR-125b2	NSun2	PAR2 activation → m6A-modified pre-miR-125b2 in a Nsun2-dependent manner ↑ → miR-125b2 ↓ → Gab2 ↑	Promotes cancer cell migration	Colorectal cancer, lung cancer	[65]
miR-126	METTL14	METTL14 ↓ → m6A-modified pri-miR-126 ↓ → pri-miR-126 ↑, miR-126 ↓	Enhances cell metastasis	Liver cancer	[83]
miR‐589-5p	METTL3	METTL3 ↑ → m6A-modified pri‐miR‐589 ↑ → pri-miR-589 ↓, miR‐589‐5p ↑	Promotes cell viability, migration and invasion	Liver cancer	[84]
miR-194–2 and miR-532	ALKBH5	ALKBH5 ↑ → m6A-modified pri-miR-194 ↓ → miR-194–2 ↓ → RAI1 ↑ → regulates the Hippo pathway	Inhibits cell growth and motility	Esophageal cancer	[85]
miR-99a	METTL14	METTL14 ↓ → m6A-modified pri-miR-99a ↑ → miR-99a ↑ → TRIB2 ↓	Promotes cancer stem cell persistence and radioresistance	Esophageal cancer	[86]
miR-92b	METTL3	Deoxycholic acid treatment → METTL3 dissociation from METTL14 and WTAP complex → m6A-modified pri-miR-92b ↓ → miR-92b ↓ → PTEN ↑ → activates PI3K/AKT signaling	Enhances cell proliferation	Gallbladder cancer	[87]
miR-19a	METTL3	METTL3 ↑ → m6A-modified pri-miR-19a ↑ → miR-19a ↑ → BAMBI ↓	Facilitates nasopharyngeal carcinoma cell proliferation and invasion	Nasopharyngeal carcinoma	[88]
miR-126-5p	METTL3	METTL3 ↑ → m6A-modified pri-miR-126-5p ↑ → miR-126-5p ↑ → PTEN ↓ activates PI3K/Akt/mTOR pathway	Promotes cell proliferation, migration and invasion and inhibits apoptosis	Ovarian cancer	[89]
miR-1246	METTL3	METTL3 ↑ → pri-miR-1246 ↓, miR-1246 ↑ → CCNG2 ↓	Promotes the proliferation and metastasis and inhibits apoptosis	Ovarian cancer	[90]
miR-25-3p	METTL3	Cigarette smoke stimulation → METTL3 ↑, with NKAP as a reader → m6A-modified pri-miR-25 ↑ → pri-miR-25↓, pre-miR-25 ↑, miR-25-3p ↑	Promotes pancreatic cancer cell proliferation, migration and invasion	Pancreatic cancer	[91]
miR-30d	YTHDC1	YTHDC1 ↓ → pri-miR-30d ↑ → miR-30d ↓ → RUNX1 ↑ → SLC2A1 ↑, HK1 ↑	Promotes aerobic glycolysis to potentiate tumor proliferation, metastasis and angiogenesis	Pancreatic cancer	[92]
miR-222-3p	METTL3	METTL3 ↑ → m6A-modified pri-miR-222-3p ↑ → miR-222-3p ↑ → STK4 ↓	Promotes tumor growth and metastasis	Thyroid carcinoma	[93]
miR-143-3p	KIAA1429 and ALKBH5	KIAA1429 ↓, ALKBH5 ↑ → m6A-modified pri-miR-143-3p ↓ → pri-miR-143-3p ↑, miR-143-3p ↓ → DDX6 ↑	Suppresses human aortic smooth muscle cell proliferation, promotes human aortic endothelial cell apoptosis and facilitates aortic dissection progression	Aortic dissection	[94]
miR-19a	METTL14	METTL14 ↑ → m6A-modified pri-miR-19a ↑ → miR-19a ↑	Promotes cardiovascular endothelial cell proliferation and invasion	Atherosclerosis	[95]
miR-25-3p	METTL3	METTL3 ↓ → miR-25-3p ↓ → PTEN ↑ → phosphorylated Akt ↓	Attenuates high-glucose induced retinal pigment epithelium cell pyroptosis	Diabetic retinopathy	[96]
miR-93	METTL3	Cigarette smoke stimulation → METTL3 ↑→ m6A-modified pri-miR-93 ↑ → pri-miR-93 ↓, pre-miR-93 ↑, miR-93 ↑ → miR-93 endocytosed from bronchial epithelial cells into macrophages through extracellular vesicle trafficking ↑ → DUSP2 ↓ → MMP9 ↑, MMP12 ↑, activates JNK pathway	Induces elastin degradation	Emphysema	[97]
miR-126	METTL3	METTL3 ↓ → m6A-modified pri-miR126 ↓ → pri-miR126 ↑, miR126 ↓	Facilitates the migration and invasion of human endometrial stromal cells	Endometriosis	[98]
miR-365-3p	METTL3	METTL3 ↑ → m6A-modified pri-miR-365-3p ↑ → pri-miR-365-3p ↓, miR-365-3p ↑	Produces pain-related behaviors and neuronal sensitization in naive mice	Inflammatory pain	[99]
miR-34-5p	METTL14	METTL14↑ → pri-miR-34 ↓, pre-miR-34 ↑, miR-34a-5p ↑ → SIRT1↓	Promotes cell cycle arrest and senescence	Intervertebral disc degeneration	[100]
miR-221/222	METTL3	Angiotensin II stimulation → METTL3 ↑ → m6A-modified pri-miR-221/222 ↑→ miR-221/222 ↑ → DKK2 ↓ → activates Wnt/β-catenin signaling	Promotes Ang-II-induced cardiac hypertrophy	Myocardial hypertrophy	[101]
miR-143	METTL3	METTL3 ↓ → m6A-modified pri-miR-143 ↓ → pri-miR-143↑, pre-miR-143 ↑, miR-143 ↓ → Yap ↑, Ctnnd1 ↑	Promotes cardiomyocyte proliferation and endogenous cardiac repair after myocardial infarction	Myocardial infarction	[102]
miR-17-3p	METTL3	METTL3 ↑ → increases binding between DGCR8 and pri-miR-17-3p → miR-17-3p ↑	Ameliorates hypoxia-induced decrease in myoproliferative capacity and increase in apoptosis	Myocardial infarction	[103]
miR-150	METTL3	METTL3 ↓, with YTHDF2 as a reader → m6A-modified pri-miR-150 ↓ → miR-150 ↓ → BDNF ↓	Increases the severity of neuropathic pain	Neuropathic pain	[104]
miR‐21	METTL3	METTL3 ↑, with HNRNPA2B1 as a reader → m6A-modified pri‐miR‐21 ↑ → miR‐21‐5p ↑ → activates the SPRY1/ERK/NF‐kB signaling pathway	Promotes inflammation and the development of obstructive renal fibrosis	Obstructive renal fibrosis	[105]
miR-320a-3p	ALKBH5	ALKBH5 ↑ → m6A-modified pri-miR-320a-3p ↓ → pri-miR-320a-3p ↑, miR-320a-3p ↓ → FOXM1↑	Promotes lung fibroblast activation and silica-induced pulmonary fibrosis	Silica-induced pulmonary fibrosis	[61]
miR-335	METTL3	METTL ↑ → m6A-modified pri-miR-335 ↑ → pri-miR-335 ↓, pre-miR-335 ↑, miR-335 ↑→ Erf1 ↓	Promotes stress granule formation and reduces the level of apoptosis in neurons and other cells	Acute ischemic stroke	[106]

In addition to its functions with methylation readers, writers and erasers, m6A modification may also facilitate miRNA maturation by promoting Dicer splicing of precursor miRNAs (Fig. 2C). In the context of non-small cell lung cancer, METTL3 has been shown to increase pre-miR-143-3p splicing in an m6A-dependent manner to promote miR-143-3p biogenesis, leading to lung cancer invasion and angiogenesis through a mechanism involving dysregulation of vasohibin 1 (VASH1) expression [62]. This finding provides a potential avenue of investigation through which to develop novel treatments for patients with non-small cell lung cancer, as well as brain tissue metastasis by cancer cells.

Interestingly, in the absence of changes to its primary transcript, it has been reported that levels of several mature miRNAs are decreased within cells after downregulation of the m6A demethylase known as FTO, and this suggests that m6A can negatively regulate miRNA biogenesis [63]. Consistent with this observation, NSUN2 methyltransferase inhibits processing of pri-miR-125b to miR-125b, and this results in a decrease in miR-125b expression levels [64]. This process of NSUN2-dependent miR-125b downregulation may be facilitated by the actions of the protease activation receptor 2 (PAR2) and can ultimately promote rectal cancer metastasis through dysregulation of the expression of GRB2-associated binding protein 2 (*Gab2*) gene [65]. Contrastingly, in studies of endocrine-resistant breast cancer cells, HNRNPA2/B1 appears to play a more complex role in miRNA biogenesis. HNRNPA2/B1 is found to be overexpressed in endocrine-resistant breast cancer cells, and this leads to upregulation of miR-1266-5p, miR-1268a and miR-671-3p, as well as reductions in levels of miR-29a-3p, miR29b-3p and miR-222 which, collectively, is linked to a reduction in the sensitivity of such cells to cancer drugs 4-hydroxytamoxifen and fulvestrant [66]. Thus, while m6A methylation is important to miRNA homeostasis and in the context of cancer, the underlying mechanisms remain to be better characterized. Since m6A methylation signatures can regulate mRNA degradation, inhibition of miRNA processing by m6A could be explained by a mechanism involving downregulation of the mRNA expression of protein factors that read, process and interact with m6A RNA species, such as DGCR8, Drosha and Dicer. Further to this issue, Chen and colleagues suggest that m6A modification on pri-miRNAs may be selectively recognized by specialized readers involved in regulating miRNA instability or degradation, or both. Evidence for such a mechanism has been demonstrated for several ncRNAs and mRNA [67, 68].

Furthermore, m6A can be found in mature miRNAs [69]. However, the origin of m6A on mature RNAs and its impact is poorly understood (Fig. 2D).

Taken together, m6A modification is essential to miRNA processing, and our understanding of the functional impact of m6A modification of miRNAs remains to be better clarified.

### 2′-O-methylation (Nm)

**Fig. 3:**
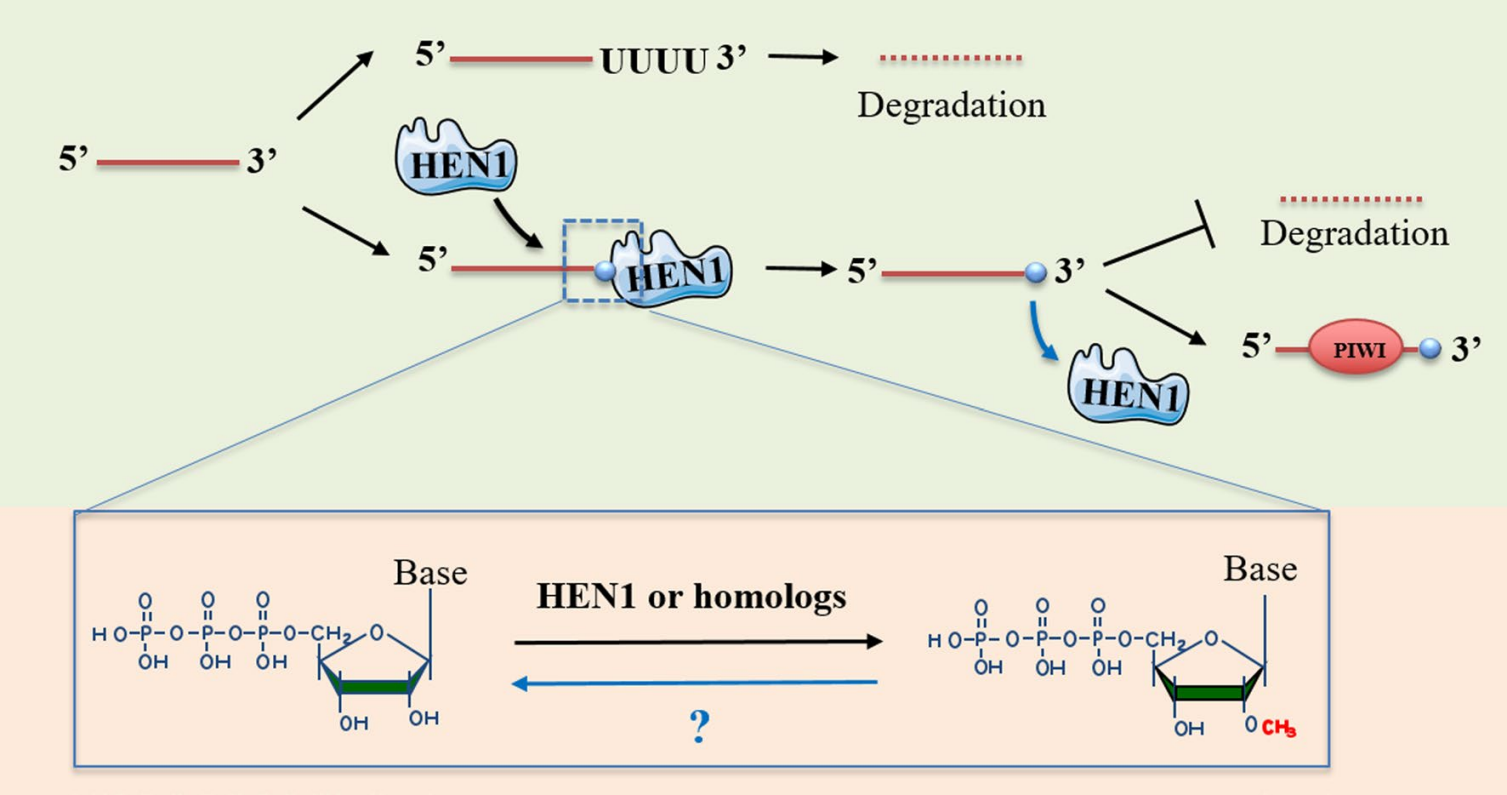
The 2′-O-methylation (Nm) modification in piRNA. HEN1 catalyzes Nm modification of piRNA, replacing a hydrogen (–H) atom on the ribose fraction 2′-hydroxyl (–OH) with a methyl-group (–CH3). During piRNA maturation, the 3′-terminal nucleotide undergoes Nm modification, leading to formation of a stable piRNA structure that is protected from 3′-uridine degradation

Another RNA modification is 2′-O-methylation (Nm), which is an abundant and highly conserved modification that replaces hydrogen (-H) atom on the ribose fraction 2′-hydroxyl (-OH) with a methyl group (-CH3) [107]. The Nm modification can be found at various sites within transfer RNAs (tRNAs), ribosomal RNAs (rRNAs), small nuclear RNAs (snRNAs) [108] and messenger RNAs (mRNAs) [109]. Also, Nm modifications are detected on the 3′-ends of small RNAs such as miRNAs and siRNAs in plants [110, 111], Argonaute 2 (AGO2)-loaded siRNAs and miRNAs in flies, as well as piRNAs in animals [112, 113]. During the maturation process of small RNAs in humans, small RNAs undergo Nm modification at their 3′ end nucleotides after their processing by Dicer or PIWI proteins [114], and this modification is important for them to form stable structures protected from 3′–5′ truncation and 3′-uridine-triggered degradation (Fig. 3) [115–118]. In addition, Nm is postulated to affect the stability of small RNAs by affecting thermodynamic properties such as base stacking and structural rigidity [119, 120].

The first identified 2′-O-methyltransferase responsible for Nm modification of small RNAs was the HUA ENHANCER 1 (HEN1) protein, discovered in *Arabidopsis* as a methylase for miRNAs and siRNAs [111, 121]. Subsequently, HEN1 homologs were found in other plants, as well as homologs that methylated piRNAs in animals, as well as AGO2-associated small RNAs in *Drosophila* [112, 122–127]. Studies have shown that *HEN1* knockout or mutation in *Arabidopsis* leads to elevated levels of heterogeneous 3′-ends and poly-U, both features of which are known to disrupt RNA stability [110, 118], and which result in aberrant lengths as well as decreased levels of small RNAs, respectively. This phenotype is predicted to be associated with the role of enzymes responsible for 3′ uridylation of small RNAs, such as HEN1 SUPPRESSOR1 (HESO1) [116, 117] and UTP:RNA uridylyltransferase 1 (URT1) [128]. For example, in *Drosophila*, loss of Pimet (HEN1 homolog) activity leads to deletion of Nm in piRNA and siRNA [129]. In addition, mutations in the *HEN1* gene accelerate neurodegeneration and shorten lifespan, suggesting that Nm of small RNAs may affect age-related signaling events within cells [130]. In zebrafish, the absence of Hen1 (HEN1 homolog) results in a decrease in piRNA content within oocytes and a shortening of exonuclease-mediated piRNAs, which ultimately leads to oocyte loss and infertility [124]. Collectively, these findings highlight the roles for HEN1 and its orthologs across a broad range of plant and animal model systems in stabilizing germline small RNAs, with species-specific consequences.

In mammals, the HEN1 homolog, HENMT1, plays an essential role in fertility. For example, in mice, loss of HENMT1 expression leads to piRNA instability, observed as reductions to piRNA volume and length, as well as developmental arrest of germ cells during the process of spermatogenesis. Particularly, loss of HENMT1 and the associated loss of piRNA collectively lead to defective meiosis and precocious and selective expression of haploid transcripts during meiosis [131]. This finding shows that HENMT1 is critical to piRNA homeostasis in maintaining TE inhibition and spermatogenesis in germ cells [131]. Of note, Nm modifications have also recently been detected in mature miRNAs in mammals. Different 3′-terminal Nm patterns of miRNAs, particularly miR-21-5p, have been reported in RNAs from non-small cell lung cancer cells from human subjects, as well as within cells of their paired normal tissue extracts. This methylation is reported to enhance the capacity for miR-21-5p to resist degradation by the polyribonucleotide nucleotidyltransferase enzyme PNPase 1 (PNPT1) which, in turn, leads to their prolonged loading onto AGO2 to form a complex that enhances the inhibition of expression of programmed cell death 4 (PDCD4) [132]. This suggests that Nm modification of miRNA can enhance miRNA stability and prevent their degradation by enzymes such as PNTP1 [132].

In addition to HEN1, other 2′-O-methyltransferases, such as FTSJ1 and FBL, are also crucial for Nm modification on small RNAs and their functions. For instance, studies have shown that the reduction of human FTSJ1 orthologs-mediated tRNA Nm modification in *Drosophila* leads to small RNA pathway dysfunction and increased susceptibility to RNA virus infection. This phenomenon is also associated with small RNA-induced gene silencing pathways [133]. FTSJ1-mediated Nm modification on tRNA also can effectively suppress DRAM1 expression, consequently inhibiting the progression of non-small cell lung cancer [134]. Yi et al. discovered that EZH2 has a direct interaction with FBL, a 2′-O-methyltransferase, leading to an enhancement in the 2′-O-methylation of rRNA, which promotes the assembly of box C/D small nucleolar ribonucleoproteins and facilitates tumor cell translation [135]. Overall, Nm modifications on tRNA play important roles in RNA silencing, translation and antiviral defense.

### 5-Methylcytidine (m5C)

**Fig. 4:**
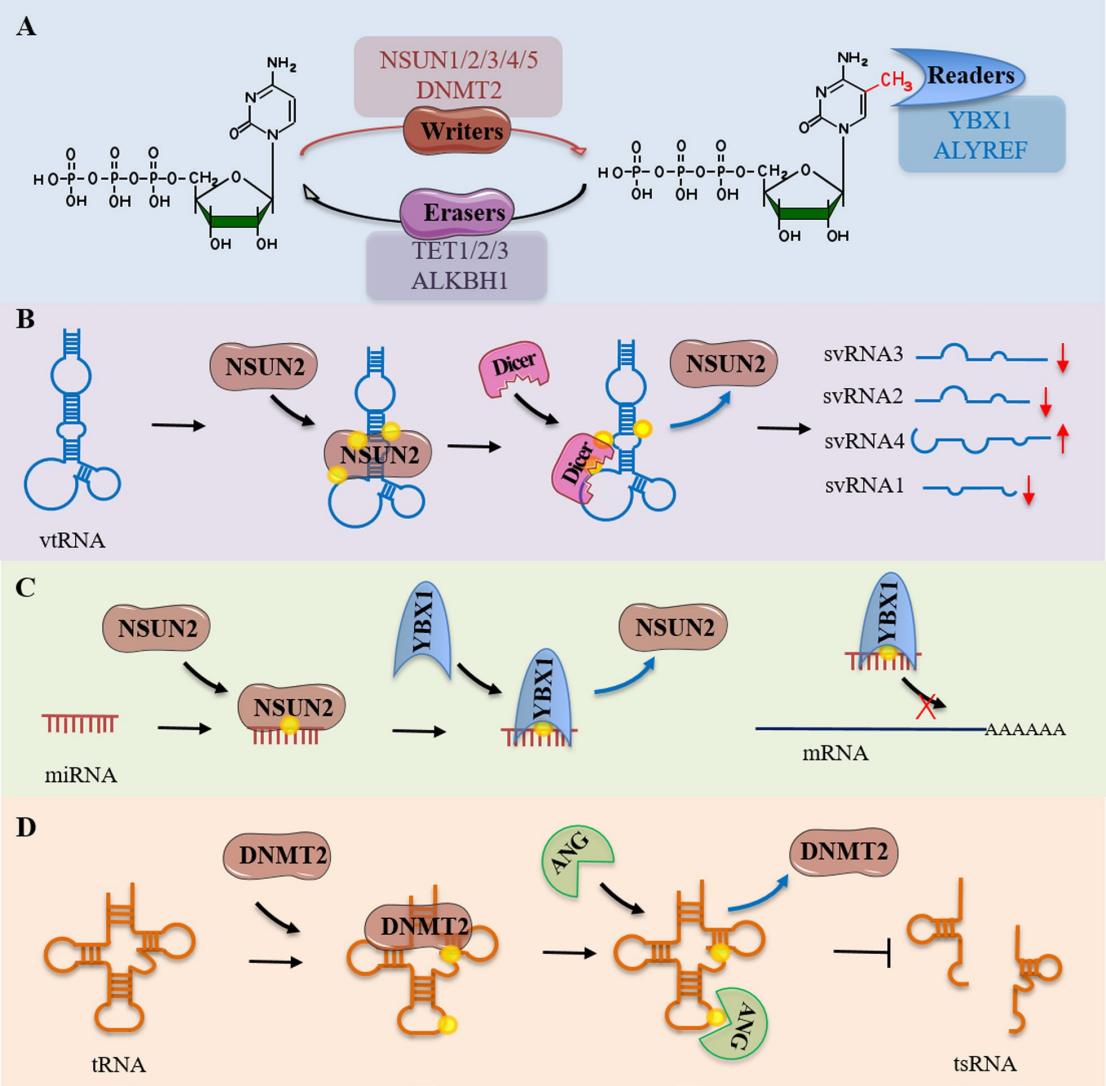
The m5C modification in small noncoding RNAs. A. The chemical structure of cytosine and the site of methylation on C5 are both shown alongside the relevant enzymes (writers, eraser and readers). B. The m5C modification in vtRNA. The m5C modification of vtRNA catalyzed by NSUN2 affects cleavage by Dicer, resulting in persistence of svRNA4 products and a relative decrease in svRNA1, svRNA2 and svRNA3 (↓ denotes downregulation, while ↑ denotes upregulation). C. The m5C modification in miRNAs. Nsun2-mediated m5C modification in miRNA can interfere with the formation of miRNA;mRNA pairing, resulting in the loss of miRNA-mediated gene silencing activity. D. The m5C modification in tRNA. DNMT2-mediated m5C modification of tRNA can reduce the affinity of ANG to tRNA, leading to a reduction in tsRNA synthesis in cells

Another RNA modification is 5-Methylcytidine (m5C) in which the fifth carbon atom (C) of cytosine is methylated within RNAs [108]. The highly conserved NSUN (NOL1/NOP2/Sun) domain family has been identified as specific enzymes responsible for m5C RNA modification [136, 137]. Also, the enzyme DNA methyltransferase 2 (DNMT2) has been found to catalyze the formation of m5C at position C38 of tRNAs (Fig. 4A) [136, 137].

All these methyltransferases can also methylate small RNAs. For example, Shobbir Hussain and coworkers found that vault RNAs, a class of small RNAs of approximately 88 to 100 nucleotides in length and transcribed by RNA polymerase III, contain 6 methylated cytosines by NSUN and formed riboprotein granules called Vault with proteins [138]. The authors also used NSUN2-deficient patient cells to further demonstrate that loss of cytosine-5 methylation in vt-RNA leads to abnormal processing of argonaute-associated small RNA fragments that could function as miRNAs (Fig. 4B) [139]. The methylation of cytosine 69 in vtRNA occurs frequently in human cells and is jointly regulated by NSUN2 and serine/arginine splicing factor 2 (SRSF2), such that vtRNA processing produces different small-vault RNA (svRNA), which is implicated in the regulation of epidermal differentiation [140]. In addition, m5C modification is also present on miRNAs [141].m5C modifications on miRNAs have been reported to interfere with the formation of miRNA/mRNA pairing, resulting in loss of gene silencing activity of the miRNAs themselves (Fig. 4C). For example, m5C modification abolishes the capacity for miRNA-181a-5p to function as a tumor suppressor and correlates with poor prognosis in glioblastoma patients [142]. The m5C modification of miRNAs is also found to result in structural changes in the RNA-induced silencing complex (RISC). For example, m5C modification at position 9 of miR-200c-3p adjacent to its recognition with the RISC complex can disrupt the hydrogen bond formed between miRNA and AGO Ser220, resulting in guanine interaction at miRNA position 8 with Arg761 of AGO translocation [69].

In addition, m5C modifications on tRNAs affects the generation of tsRNA species. The formation of tsRNAs is reported to be induced in response to stress, since unconventional 5′ initiation sites can be found in the 5′ UTR of stress response transcripts to inhibit canonical translation and favor ribosome assembly [143]. Thus, m5C modification on tRNA protects them from angiogenin (ANG) processing into tsRNA (Fig. 4D) [144–148]. In contrast, DNMT2-mediated loss of cytosine 38 methylation of tRNA leads to accumulation of tsRNAs, leading to mis-translation of specific codons and disruptions to protein synthesis [146]. These tRNA and tsRNA alterations therefore inhibit protein synthesis and negatively affects cell function and development [144, 146, 147, 149]. Furthermore, in sperm, small RNAs can encode paternal information through m5C modifications with the capacity for intergenerational transmission of paternally acquired phenotypes. For example, Zhang and colleagues altered the expression profile of sperm small RNAs, including the levels of tsRNAs and rRNA-derived small RNAs, by knocking out mouse tRNA methyltransferase DNMT2. This intervention led to a prevention of the high-fat diet-induced elevation of RNA modifications (m5C, m2G) in the 30-40nt RNA fraction in sperm and subsequent abolishment of the transmission of small RNA-mediated metabolic disorders from high-fat diet-induced sperm. This finding suggests that DNMT2-mediated m5C modification of RNAs contributes to the secondary structure and biological characteristics of small RNAs that underlie their capacity for paternal epigenetic memory, programmed as "coding marks" within sperm RNAs [150].

### Other modifications on small RNAs

In addition to the modifications mentioned above, various other small RNA modifications have also been reported, including pseudouridine (Ψ) modifications, m7G modifications and m1A modifications, and of which also seem to play important roles in cell biological processes, as described below. One caveat is that the underlying effects of such modifications on the structure and functions of RNAs remain to be better characterized.

#### Pseudouridine (Ψ)

Pseudouridine (Ψ), an isomeric form of uridine also known as 5-ribosyluracil, was discovered in the 1950s and is described as the most abundant type of post-transcriptional RNA modification discovered in all kingdoms of life [151–153]. Pseudouridine is catalyzed by an evolutionarily conserved family of pseudouridine synthases (PUS) or by RNA-dependent mechanisms that involve a significant number of H/ACA box small nucleolar RNA (snoRNA) [154]. Pseudouridine can be detected in tRNAs [155], rRNAs [156, 157], small nuclear RNAs (snRNAs) [158–160] and mRNAs [161, 162]. Its presence is known to affect the biogenesis, structure, function and coding potential of RNA, with attendant effects on downstream signaling and cell homeostasis.

Recent studies have found that pseudouridine catalyzed by PUS7 could activate tsRNAs that are involved in protein synthesis which are vital to the functions of mammalian stem cells. For example, inhibition of a pseudouridine-driven regulatory network can severely affect hematopoiesis and promote the incidence and pathogenesis of human myeloid malignancies [163]. With regard to the impact of pseudouridine synthesis on miRNAs, it has been reported that inhibition of PUS10 results in reduced mature miRNA and accumulation of primary miRNA, yet this process is independent of catalytic activity of ubiquitin-specific peptidase 10 (USP10) [164]. In another example, endogenous TruB1, a predominant mammalian pseudouridine synthase, is able to bind the stem-loop of pri-let-7 to enhance the interaction of this miRNA with the microRNA processor protein DGCR8, so as to enhance the maturation of let-7 microRNA family members that signal to inhibit cell proliferation [165]. These studies suggest that ability for cells to synthesize pseudouridine, as well as the presence of pseudouridine on multiple small RNA species are all critical to the regulation of critical biological functions.

#### N7-methylguanosine (m7G)

N7-methylguanosine (m7G) is a methylated modification of the seventh nitrogen atom of guanine in RNA, and the modification is documented in tRNAs, rRNAs, mRNAs and miRNAs [166–170]. The m7G modification within cells can be catalyzed by METTL1 and WD repeat domain 4 (WDR4) proteins [171]. For example, METTL1 binds directly to the miRNA precursor via m7G and accelerates miRNA maturation [168]. After pre-miRNA processing, m7G can persist on mature miRNAs and its presence can influence the function of mature miRNAs. For example, in lung cancer cells, a mature let-7e miRNA that encodes a m7G modification is capable of downregulating the stability and translation efficiency of the mRNA transcript encoding high mobility group protein 2 (HMGA2). This, in turn, leads to a reduction in levels of translated HMGA2 protein and subsequent arrest of lung cancer cell proliferation and migration [168].

#### N1-methyladenosine (m1A)

N1-methyladenosine (m1A) modification involves the methylation of the first nitrogen atom of adenine within RNA. This modification is mainly detected in tRNAs, rRNAs, mRNAs and small RNAs, and its presence has been found to influence the structure and functions of these RNA species [172–174]. Particularly, small RNAs can regulate gene expression through m1A. For example, Su and colleagues found that TRMT6/61A, an RNA methylase enzyme, was highly expressed in urothelial carcinoma cells derived from human bladder, compared with normal cells, and this was accompanied by higher levels of m1A modification across multiple RNAs including tsRNAs. This resulted in dysregulation of the tsRNA targetome and contributed to cellular functions related to malignant transformation, including a direct effect on the unfolded protein response within these cancer cells [175].

## Clinical applications

### Prevention

Current evidence in the literature has indicated that therapeutic manipulation of small RNA modification may be clinically beneficial in the treatment and prevention of human disease conditions. For example, Wang and colleagues investigated changes in methylation of 22 miRNAs in 57 cases of human neural tube defects (NTDs) and reported that methylation of the microRNA hsa-let-7 g directly effects its expression (176). Furthermore, the methylation levels for hsa-let-7 g were significantly correlated with folate concentration. Thus, the correlation between aberrant methylation of hsa-let-7 g and folate metabolism could indicate that, by improving early-pregnancy nutrition, NTDs could be avoided through a mechanism which involves adequate dietary supply of methyl-donors for miRNA modification within fetal cells [176]. In addition, growing evidence indicates that small RNAs in sperm can mediate the intergenerational transmission of paternally phenotypes [177, 178]. For example, Chen and coworkers found that small RNA modifications in sperm were involved in encoding paternity information [150]. There, the authors found that depletion of mouse tRNA methyltransferase DNMT2 prevented the high-fat diet (HFD)-induced elevation of RNA modifications (m5C, m2G) in the 30-40nt RNA fraction of sperm, and this blocked the epigenetic transmission of phenotypic traits for HFD-induced metabolic disorder that would have otherwise been detected in the offspring. Indeed, Dnmt2-mediated m5C modifications is crucial to endow small RNAs within sperm with specialized secondary structures and biological properties, and this is a powerful example of how small RNA modifications are critical to the epigenetic inheritance of paternal traits [150]. Thus, in these examples of NTD and sperm function, small RNA modifications influence the developmental homeostasis and organismal survival of mammalian offspring.

### Prediction and surveillance

In 2006, Seidel and colleagues applied a chromatography method to discover that the modified nucleosides in urine samples have high sensitivity and specificity as potential biomarkers to identify a variety of cancers in patients [179]. Recently, with the rapid development of detection techniques, the great potential of small RNA modification as a biomarker of disease is being realized. For example, Su and workers used an improved RNA sequencing detection technique of Thermostable Group II Intron Reverse Transcriptase (TGIRT) and discovered the presence of m1A, m1G and N2, N2- dimethylguanosine (m^2^_2_G) modifications on tsRNAs and rsRNAs in bladder cancer cells [180]. Yan and colleagues applied a highly efficient liquid chromatography-tandem mass spectrometry method and discovered that small RNA modifications in the liver cells of diabetic mice were significantly altered compared to control treatment, highlighting the correlation between small RNA modifications and diabetes [181]. Zhang and coworkers found that 2′-O-methylcytidine (Cm), m7G, 2′-O-methylguanosine (Gm), and m^2^_2_G modifications in 15–25 nt RNA from cells within the cerebral cortex of Alzheimer's disease patients were significantly increased, compared with normotypic control samples [182]. Konno and colleagues reported that methylation of some miRNAs was elevated in tumor samples compared to normal tissues. Of note in one particular study, the methylation levels for miR-17-5p in serum were sensitive enough to distinguish patients with pancreatic cancer from healthy individuals [69]. Furthermore, small RNA modifications have been suggested to be reliable as a potential biomarker for male. There, studying RNA samples from patients with asthenozoospermia and teratozoospermia relative to controls, Guo and colleagues used a high-throughput sequencing platform to detect RNA modifications and identified 13 RNA modification signatures on total sperm RNA, as well as 16 RNA modification signatures on sperm RNA fragments of varying sizes. Particularly, the modifications m1G, m5C, m2G and m1A were found to be significantly correlated with clinically-graded sperm motility measures [183].

In addition to their potential value as diagnostic markers, the presence and relative abundance of small RNA modifications may also serve as prognostic biomarkers. For example, in patients diagnosed with glioma, low miRNA-181a-5p expression and cytosine methylation levels were associated with poor survival prognosis (reported as median survival rates of 12.4 months and 8.5 months, respectively), while glioma patients with high levels of unmethylated miRNA-181a-5p were found to have a better survival prognosis (median 16.5 months) [184]. Guzzi and colleagues reported that the dysregulation of the terminal oligoguanine (TOG) at 5′-terminal end of tRFs, which is regulated by pseurouridine driven by PUS7 activity, is linked to leukemia transformation and reduced survival rates in patients and increases the risk of progressing from myelodysplastic syndrome (MDS) to acute myeloid leukemia [185].

Taken together, these findings suggest that small RNA modifications may be informative as markers that reflect the pathogenesis and progression of human disease.

### Therapy and other applications

Given the examples of direct effects for small RNA modifications on human diseases such as bladder cancer and armed with the knowledge that evolutionarily conserved mechanisms drive small RNA modifications within cells, researchers are now exploiting these discoveries to design novel RNA-based treatments for human disorders [186]. For example, it has been found that Nm modified siRNAs are significantly more stable in serum so that it persists longer as an effective treatment to inhibit Enterovirus Type 71 (EV71) replication [187]. In another example that demonstrates their stability when delivered into animals, siRNAs modified by thiophosphate, Nm and other modifications, stimulant-related analytes were delivered intravenously into rats and, 24 h later, these modified RNA species could still be detected in rat blood and urine samples by liquid chromatography-high-resolution/high-precision mass spectrometry [188]. Nm modifications in plant miRNAs can extend their half-lives, and so this must be taken into consideration regarding the use of modified RNAs, their potential to be ingested by humans and the impact of RNA treatments in plant horticulture that has consequences on human physiology in those that adopt predominantly plant-based diets [189, 190]. In a related example, the tRNA methyltransferase known as TrmH which is required for G18 tRNA Nm, is not present in most bacteria, however, specific Nm modification to guanosine in bacterial tRNA position 18 is required to inhibit Toll-like receptor 7-mediated immune activation during a human host–pathogen response episode [191, 192]. Thus, Nm modification may be a feature of active selection in symbiotic and pathogenic species, such as in regulating the recognition of autologous and non-autologous-derived RNAs [15].

**Fig. 5:**
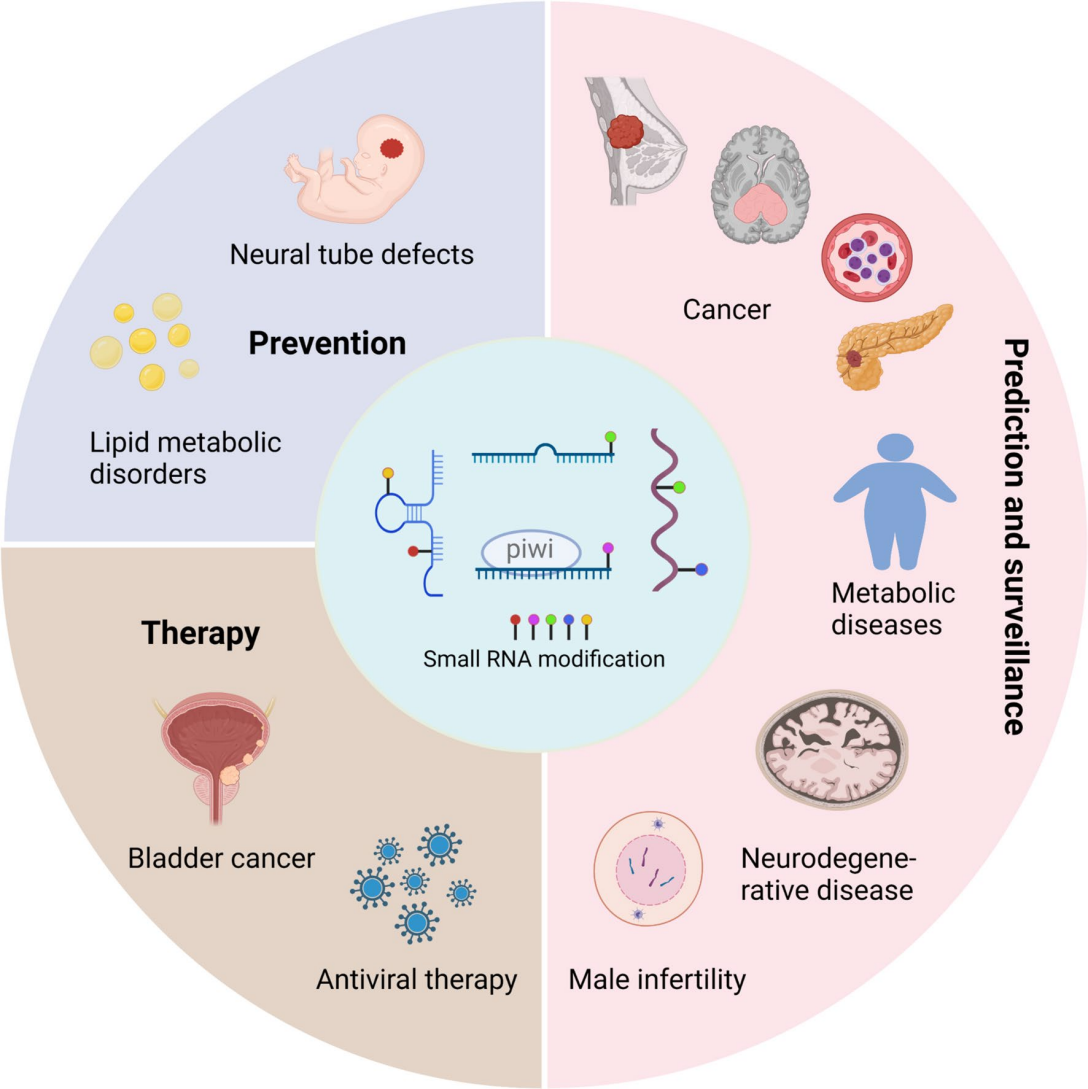
Applications of small RNA modifications. Current applications of small RNA modifications are described in the context of disease prevention (such as in neural tube defects (NTDs), lipid metabolic disorders), cancer treatment (such as bladder cancer), disease prediction and surveillance (such as for cancer, metabolic diseases, neurodegenerative disease and male infertility). This figure was created with BioRender.com

Recently, as indicated above, Su and colleagues reported that m1A modifications are highly enriched in 22-nucleotide long 3′ tRNA fragments and is dependent on its methylase TRMT6/61A [193]. In bladder cancer cells, high TRMT6/61A expression is observed, m1A modification levels of tRFs is increased and these molecular findings correlated with abnormal regulation of tRF target genes, such as those critical to the unfolded protein response [193]. Thus, small RNAs can regulate gene expression through base modifications, and this highlights their potential as a therapeutic avenue for the design of treatments to conditions such as bladder cancer (summarized in Fig. 5).

## Detection techniques

### Detection techniques of small RNAs

As researchers discover the critical roles for small RNAs in physiological and pathological processes, an increasing number of sequencing techniques to detect small RNAs with high sensitivity and specificity have been reported. However, the complex landscape of small RNA modification presents as a challenge for high-throughput analysis of small RNAs because such modifications interfere with the preparation of RNA-seq libraries and can limit their detection. Table 3 lists the current approaches to improving small RNA sequencing by overcoming specific RNA modifications.

**Table 3:** Detection techniques of small RNAs

Method	Verified small RNA species	Specific features of the approach	References
ARM-seq	tRNA, tsRNA	AlkB treatment to remove m1A, m3C and m1G modifications in tRNA	[194]
DM-tRNA-seq	tRNA	AlkB treatment to remove m1A, m3C and m1G modifications in tRNAs; thermostable group II intron RT (TGIRT) with high processivity to generate cDNA from highly structured tRNA adds RNA-seq adaptors by template-switching without RNA ligation	[195]
multiplex small RNA-seq library preparation method (MSR-seq)	tRNA, tsRNA and other small RNAs	Design of a biotinylated oligonucleotide used for barcode adapter ligation, immobilization, on-bead reverse transcription, second adapter ligation and PCR; AlkB treatment removes m1A and m1G modifications in tRNAs	[196]
CPA-seq	small RNAs including tsRNA, snsRNA, snosRNA, lncsRNA, miRNA	Use of a deacylation buffer (pH = 9.0) to remove aminoacyl residues in aminoacyl-tRNA-derived 3′-tsRNAs; Cap-Clip to remove the 5′-cap and 5′-ppp from RNAs to generate 5′-P termini; T4 PNK to reduce terminus multiplicities; AlkB and AlkB(D135S) (AlkB mix) used to remove methylation in m1A, m3C and m1G; TGIRT-III, a highly processive reverse transcriptase, used to increase the detection of sRNAs derived from tRNAs containing m1A, m3C and m1G sites	[197]
PANDORA-seq	miRNA, tsRNA and rsRNA	AlkB treatment to remove m1A, m3C, m1G and m_2_^2^G modifications in tsRNAs;T4PNK treatment to convert 5′-OH at the 5′end into 5′-P and 3′-P and 2′,3′- cP at the 3′end into 3′-OH	[198]
AQRNA-seq	all types(tRNA and miRNA, mRNA, rRNA, etc.)	AlkB treatment to remove m1A, m1G and m1I modifications; Shrimp alkaline phosphate treatment to convert 5′-P into 5′-OH and 3′-P into 3′-OH; Adaptor ligation at the 3′end of RNAs, to resolve the issue of 5′terminal modification	[199]
cP-RNA-seq	5′-tRNA halves; cP-containing RNA repertoires in various transcriptomes	Gel-purified RNAs specific sizes are purified and treated with a phosphatase (CIP), followed by treatment with a periodate (NaIO4) to disrupt 3′-ends of RNAs containing 3′-P and 3′-OH ends; T4PNK to selectively capture RNAs with 2′,3′-cP at their 3′ termini	[200]
5´XP sRNA-seq	miRNA, piRNA, tsRNA and rsRNA	Simultaneous capture of 5′-P and non-5′-P RNAs with the 5′-P RNA tagged with a barcode sequence resolved during bioinformatic analyses	[201]

### Detection techniques of small RNA modifications

Several RNA-seq methods involve sequencing of the cDNA intermediate of RNA, and the conversion of RNA to cDNA can lead to loss of detection of small RNA modification. Table 4 lists the currently reported detection methods for small RNA modification sequencing. Recent studies have described two innovative methods for detecting m6A modifications on mRNA, including m6A-SAC-seq and eTAM-seq. The m6A-SAC-seq method uses the Dim1/KsgA family of dimethyltransferases, which transfer the methyl group from S-adenosyl-L-methionine (SAM) to adenosines, resulting in the formation of m6A, followed by N6,N6-dimethyladenosine (m62A) in consecutive methylation reactions [202]. eTAM-seq relies on global A deamination, which enables the detection of m6A as persistent A [203]. Nonetheless, neither of these two methods has been applied to detect small RNA modifications yet. With further modifications, both of these methods would be utilized in detecting small RNA modifications in the future. In summary, these technologies provide basic scientific tools and methods for comprehensive analysis of small RNA modifications and biological studies.

**Table 4:** Techniques to detect small RNA modifications

Techniques	Modification	Verified small RNAs	Principle	Characteristics (including advantages and challenges)	References
m6A-individual nucleotide resolution crosslinking and immunoprecipitation (miCLIP- m6A)	m6A	snoRNA	Mapping of m6A residues achieved through the creation of unique signature mutations using m6A-specific antibodies and UV crosslinking	Identifies the exact sites of m6A; Without pretreatment of cells with modified nucleotides; Unbiased identification of m6A residues	[204]
m5C-individual nucleotide resolution crosslinking and immunoprecipitation (miCLIP-m5C)	m5C	Vault RNA	The specific complex containing NSUN2 and m5C leads to a truncation site during RT-PCR, which can be interpreted as a marker for m5C modification	Identifies the exact sites of m5C	[139]
Demethylase tRNA sequencing (DM-tRNA-seq)	m1A, m3C, m1G, m^2^_2_G and m3U	tRNA, rRNA	Use of AlkB demethylase and its engineered mutant as central components to remove m1A, m3C and m1G modifications at the Watson–Crick face in tRNA prior to cDNA synthesis	Use of a modification index (MI) to assess the quantitative nature of each detectable modification site	[205]
Borohydride Reduction sequencing (BoRed-seq)	m7G	miRNA	RNA fragments that contain certain modifications can be enriched through specific antibody immunoprecipitation	Approach exhibits high specificity but lacks single-nucleotide resolution and is unable to detect methylation in low-abundance RNAs	[168]
RiboMeth-seq	Nm	rRNA	Nm can be mapped by analyzing the read-end information in sequencing data due to its resistance to alkaline hydrolysis	This method is capable of identifying missing peak regions that relate to Nm locations	[206, 207]
2′-OMe-seq	Nm	rRNA	Restricting the concentration of either dNTP or Mg2 + during RT reactions leads to halting of RT at Nm sites	A relatively straightforward and sensitive approach with strong specificity; allows identification at single-base resolution and quantitation of 2΄-O-methylated residues	[208]
Direct m6A Sequencing	m6A	tRNA	Use of KlenTaq DNA polymerase to function as an reverse transriptase, which can result in the incorporation of incorrect nucleotides at m6A sites	Enables the direct detection of m6A sites from untreated RNA sequencing data	[209]
RNA bisulfite sequencing technology (RNA-BisSeq)	m5C	tRNA, rRNA	Addition of sodium bisulfite deaminates unmethylated cytosines (at acidic pH) or uracil (at basic pH), preserving methylated cytosines	Provides single-nucleotide resolution avoids the requirement of high RNA concentrations for analysis; Unable to react with cytosines that are base-paired; Cannot differentiate between 5-methylcytosine and 5-hydroxymethylcytosine	[210, 211]
RBS-seq	m5C、Ψ and m1A	tRNA, rRNA	Optimizing bisulfite treatment conditions and concomitant detection of all three modifications within the same RNA	Identification of every modification through a distinct chemical method that facilitates accurate mapping of all three modifications in a single RNA molecule, thereby enabling co-variation analyses	[212, 213]
Ψ-seq	Ψ	rRNA, tRNA and snRNA	N-cyclohexyl-N′-β-(4-methylmorpholinium)-ethylcarbodiimide (CMC) can label Ψ, leading to the formation of CMC-Ψ adducts that cause RT to halt	Unbiased, quantitative profiling of Ψ across the transcriptome at the single-nucleotide resolution level	[214]
m7G Mutational Profiling sequencing (m7G-MaP-seq)	m7G	rRNA, tRNA	By reducing sodium borohydride, positions with m7G modifications are transformed into abasic sites which can be directly detected as cDNA mutations	High throughput detection of m7G modifications at single nucleotide resolution	[215]
AlkAniline-Seq	m7G and m3C	rRNA, tRNA	The resistance of m3C and m7G to NaBH4-aniline treatment and cleavage makes it possible to use selective ligation to enrich modified fragments	Does not adopt traditional RNA sequencing chemistry and depends on a chemical-based method for selectively enriching reads in the resulting libraries	[216]
Hydrazine-Aniline Cleavage sequencing (HAC-seq)	m3C	tRNA	m3C-modified sites can be selectively cleaved through treatment with hydrazine/aniline, allowing for their mapping through calculation of the cleavage ratio	Unbiased and transcriptome-wide detection of m3C RNA modification at the single-nucleotide level	[217]
HydraPsiSseq	Ψ	rRNA	Reliant on specific protection from hydrazine/aniline cleavage	Absolute measurements of modification levels; only requires extremely small amounts of RNA	[218]
LC–MS-based RNA sequencing (2D mass-tR direct RNA sequencing)	Ψ, m5C, etc	Short synthetic RNAs (< 35 nt), tRNA	Introduce a 2D hydrophobic end-labeling strategy into conventional mass spectrometry-based sequencing, which enables the de novo sequencing of RNA mixtures and improves the efficiency of sample utilization	Accurately identifies, locates and quantifies base modifications in both single and mixed RNA samples, with single-base resolution; can directly read the complete sequence; can be applied to samples containing multiple different modifications	[219]

## Conclusion and perspective

As a recently discovered class of regulatory RNAs, small RNAS (also known as sncRNAs) can negatively or positively regulate the expression of tumor markers through different molecular pathways and intracellular signaling mechanisms. These functions for small RNAs depend on their sequence, their three-dimensional structure and their extent of RNA modification. As highlighted in this review, emerging evidence suggests that RNA modifications are essential not only in their capacity to influence the biogenesis and function of small RNAs, but their potential value as biomarkers for human diseased states is also noted [15]. Novel detection technologies including high-throughput LC–MS/MS and sequencing-based approaches have accelerated our ability to identify, quantify and define the roles of small RNA modifications in homeostasis and diseases [137, 181, 195, 220, 221]. However, in contrast to the pace of the discovery of diverse types of small RNAs, which has been relatively rapid [14], research into the significance and biological impact of small RNA modification in health and disease remains in its infancy. One of the most important tasks in the future is to address these technical challenges that enable researchers in the field of sncRNA biology to capture and study all known modified sncRNA sequences with high sensitivity and specificity. It is anticipated that these advances will lead to new insights into the physiological roles of modified small RNAs and accelerate sncRNA drug discovery as viable treatments for human health conditions.

## Data Availability

Not applicable.
